# Muscle ectopic fat deposition contributes to anabolic resistance in obese sarcopenic old rats through eIF2α activation

**DOI:** 10.1111/acel.12263

**Published:** 2014-08-19

**Authors:** Nicolas Tardif, Jérôme Salles, Christelle Guillet, Joan Tordjman, Sophie Reggio, Jean-François Landrier, Christophe Giraudet, Véronique Patrac, Justine Bertrand-Michel, Carole Migne, Marie-Laure Collin, Jean-Michel Chardigny, Yves Boirie, Stéphane Walrand

**Affiliations:** 1 Clermont Université, Université d'Auvergne, Unité de Nutrition HumaineBP 10448, Clermont-Ferrand, F-63000, France; 2 INRA, UMR 1019, UNH, CRNH AuvergneClermont-Ferrand, F-63000, France; 3 UPMC, Inserm U872 Equipe 7, Centre de Recherche des CordeliersParis, F-75006, France; 4 INRA, UMR 1260Marseille, 13385, France; 5 IFR30, Inserm U563, CHU PurpanToulouse, F-31024, France; 6 CHU Clermont-Ferrand, Service de Nutrition CliniqueClermont-Ferrand, F-63003, France

**Keywords:** ceramide, eIF2α signaling, high-fat diet, muscle protein synthesis, obesity, sarcopenia

## Abstract

Obesity and aging are characterized by decreased insulin sensitivity (IS) and muscle protein synthesis. Intramuscular ceramide accumulation has been implicated in insulin resistance during obesity. We aimed to measure IS, muscle ceramide level, protein synthesis, and activation of intracellular signaling pathways involved in translation initiation in male Wistar young (YR, 6-month) and old (OR, 25-month) rats receiving a low- (LFD) or a high-fat diet (HFD) for 10 weeks. A corresponding cellular approach using C2C12 myotubes treated with palmitate to induce intracellular ceramide deposition was taken. A decreased ability of adipose tissue to store lipids together with a reduced adipocyte diameter and a development of fibrosis were observed in OR after the HFD. Consequently, OR fed the HFD were insulin resistant, showed a strong increase in intramuscular ceramide level and a decrease in muscle protein synthesis associated with increased eIF2α phosphorylation. The accumulation of intramuscular lipids placed a lipid burden on mitochondria and created a disconnect between metabolic and regulating pathways in skeletal muscles of OR. In C2C12 cells, palmitate-induced ceramide accumulation was associated with a decreased protein synthesis together with upregulated eIF2α phosphorylation. In conclusion, a reduced ability to expand adipose tissues was found in OR, reflecting a lower lipid buffering capacity. Muscle mitochondrial activity was affected in OR conferring a reduced ability to oxidize fatty acids entering the muscle cell. Hence, OR were more prone to ectopic muscle lipid accumulation than YR, leading to decreased muscle protein anabolism. This metabolic change is a potential therapeutic target to counter sarcopenic obesity.

## Introduction

Fat mass increases from 20% to 40% between ages 20 and 80 years, and lipid deposition is modified, with increased liver and skeletal muscle fat infiltration. This age-associated adiposity acts synergistically with sarcopenia, worsening disability through ‘sarcopenic obesity’. Increased lipid accumulation in many tissues is associated with the appearance of insulin resistance (Slawik & Vidal-Puig, 2007). Inversely, promoting the storage of fat preferentially in adipose tissue with adiponectin transgenic ob/ob mice or peroxisome proliferator-activated receptor gamma (PPARgamma) agonist treatment improved insulin sensitivity (Kim *et al*., 2007). Besides increasing the storage capacity of fat in adipocytes, increasing fatty acid oxidation in muscle can also prevent lipotoxicity (Henique *et al*., 2010). Very old transgenic PGC1α (peroxisome proliferator-activated receptor gamma coactivator 1 alpha) mice showed increased muscle mitochondrial activity and improved metabolic responses as illustrated by decreased circulating lipid levels and increased insulin sensitivity (Tiraby *et al*., 2007; Wenz *et al*., 2009).

The increased lipid content inside muscle in older persons is independently associated with insulin resistance (Ryan & Nicklas, 1999; Zoico *et al*., 2010), and a moderate weight loss improves muscle lipid infiltration and insulin resistance in postmenopausal women (Mazzali *et al*., 2006). Muscle fat accumulation in older people is associated not only with metabolic abnormalities, but also with reduced strength and poorer scores in performance tests and incident mobility disability (Goodpaster *et al*., 2001; Zoico *et al*., 2010). Further, the increase in intramuscular fats coincides longitudinally with the progressive muscle weakening seen in aging (Delmonico *et al*., 2009).

Thus, besides the effect of muscle fat accumulation on insulin sensitivity, muscle lipid accumulation may also promote specific changes in muscle protein metabolism. Some indirect evidence has given support to this hypothesis. Muscle protein synthesis is blunted in obese young (Guillet *et al*., 2009) and older (Katsanos & Mandarino, 2011) subjects in relation with body fatness, suggesting that increased adipose tissue mass or ectopic lipid deposition may impair skeletal muscle protein synthesis. Anderson and coworkers (Anderson *et al*., 2008b) noted a significant reduction in the ability of muscles from mice fed a high-fat diet to stimulate protein synthesis following a meal, but intramuscular lipid content was not measured. These data suggest that lipid accumulation following a chronic high-fat diet interferes with signaling pathways involved in muscle response to anabolic stimuli, blunting the activation of protein synthesis.

We hypothesized that metabolic changes in response to dietary lipids during aging were implicated in the loss of muscle mass through lipid infiltration and altered translational regulation in old rats. We first tested the impact of muscle lipid accumulation on protein synthesis rate in old sarcopenic rats fed a high-fat ‘Western’ diet. Second, we measured the effect of lipid infiltration, by modulating ceramide content, on protein synthesis rate and its translational regulation in C2C12 muscle cells.

## Results

### Aging alters the pathogenesis of diet-induced obesity

The young rats developed diet-induced obesity (DIO) with the high-fat diet, as shown by increased body weight and body fat deposits (Table1). There was no apparent difference in body weight between old control and old high-fat groups, although adipose tissue amounts were slightly increased in the old high-fat rats. Of note, the young rats became more obese than the old ones. In further support of our previous findings (Zangarelli *et al*., 2006), hindlimb muscle weight was significantly reduced by age, in particular type 2 fiber muscle weight (Table1). In addition, compared with control group, 10 weeks of high-fat diet (HFD) were responsible for a significant decrease in hindlimb muscle weight in old rats. Therefore, with both advancing age and DIO, muscle mass declined while body fat rose in rats. This inverse relationship has led to the concept of sarcopenic obesity, implying an age-dependent molecular link between body and muscle fat accumulation, insulin resistance and the decline of muscle mass.

**Table 1:** Characteristics of the rats

	YLF	YHF	OLF	OHF
Body weight (g)				
Before diets	476 ± 19	478 ± 19	625 ± 39	623 ± 33
After diets	592 ± 3*	696 ± 12***,*	637 ± 19	693 ± 33
Hindlimb muscle weight (g)	19.2 ± 0.2	19.4 ± 0.3	12.6 ± 0.9**	9.4 ± 0.8**,***
Tibialis anterior muscle weight (g)	1.99 ± 0.07	1.91 ± 0.06	0.68 ± 0.03**	0.53 ± 0.04**
Body fat depots (g)				
Intra-abdominal fat mass	11.7 ± 0.8	22.8 ± 1.9***	14.6 ± 1.3	19.9 ± 2.3**,***
Peri-epididymal fat mass	10.9 ± 0.6	19.7 ± 1.1***	11.3 ± 1.2	15.8 ± 1.8**,***
Glucose (mmol/L)	8.1 ± 0.8	9.2 ± 1.4	7.3 ± 0.5	8.8 ± 0.8
Insulin (ng/mL)	0.58 ± 0.06	1.04 ± 0.28	0.74 ± 0.26	0.56 ± 0.15
Leptin (ng/mL)	3.1 ± 0.3	5.3 ± 0.4***	3.6 ± 0.3	5.5 ± 1.0***
Adiponectin (ng/mL)	4.8 ± 0.6	4.2 ± 0.3	5.1 ± 0.4	5.8 ± 0.8
Triglycerides (g/L)	1.01 ± 0.10	0.81 ± 0.08	0.91 ± 0.17	0.99 ± 0.12
FFA (mmol/L)	0.53 ± 0.04	0.43 ± 0.04	0.43 ± 0.03	0.71 ± 0.02*****
Total cholesterol (g/L)	0.78 ± 0.07	0.62 ± 0.03	0.81 ± 0.05	0.9 ± 0.04**
sTNF-R1 (pg/mL)	234.3 ± 18.5	233 ± 14.8	307.5 ± 42.3	380.8 ± 62.8**,***
sTNF-R2 (pg/mL)	1146 ± 55	1260 ± 71	1200 ± 65	1644 ± 45**,***

Values ± SEM for eight animals per group.

YLF, young rats fed a low-fat diet, YHF, young rats fed a high-fat diet, OLF, old rats fed a low-fat diet, OHF, old rats fed a high-fat diet, FFA, free fatty acids.

* *P* < 0.05 vs. before diet

** *P* < 0.05 age effect (same diet)

*** *P* < 0.05 diet effect (same age).

**Figure 1:**
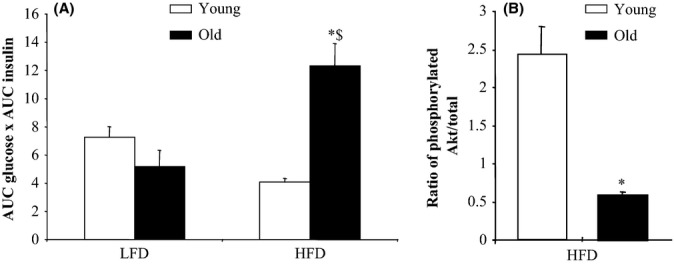
Whole-body and skeletal muscle insulin sensitivity indices in young and old rats fed a low-fat or a high-fat diet for 10 weeks. (A) Index of whole-body insulin resistance measured by intraperitoneal glucose tolerance test. Insulin resistance was estimated by the product of the area under the curve (AUC) of the glucose and the AUC of insulin after intraperitoneal glucose infusion. (B) Akt phosphorylation state in tibialis anterior muscle. Phosphorylated Akt to total Akt protein content ratio was measured by Western blot. Values ± SEM for eight animals per group. *P < 0.05 age effect (same diet), ^$^*P* < 0.05 diet effect (same age). LFD, low-fat diet; HFD, high-fat diet.

Although fasting glycemia and insulinemia were not changed, data from the intraperitoneal glucose tolerance test indicated that impaired glucose tolerance was concomitant with obesity in old rats (Table1 and Fig.1A). In old adults (25-month-old rats), the obesity in rats fed the HFD was morbid and associated with resistance to insulin, in particular in skeletal muscle as illustrated by the reduced Akt activation (Fig.1B). Consistent with these data, blood concentration of free fatty acids (FFA), cholesterol and inflammation markers, that is, cytokines, were increased considerably in old rats fed the high-fat diet (Table1).

### Diet-induced obesity promotes adipose tissue fibrosis and inflammation and muscle lipid infiltration in old rats due to altered mitochondrial activity

**Figure 2:**
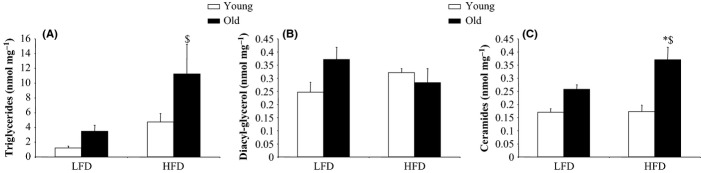
Triglyceride, diacyl-glycerol, and ceramide content in tibialis anterior muscle of young and old rats fed a low-fat or a high-fat diet for 10 weeks. Triglyceride (A), diacyl-glycerol (B), and ceramide (C) concentrations in tibialis anterior muscle were analyzed by gas-liquid chromatography after total lipid extraction. Ceramide, diacyl-glycerol (DAG) and triglyceride (TG) concentrations are expressed as nmoles/mg proteins. Values ± SEM for five animals per group. *P < 0.05 age effect (same diet), ^$^*P* < 0.05 diet effect (same age). LFD, low-fat diet; HFD, high-fat diet.

The chemical determination of the concentration of fat-reactive species in muscle tissue revealed more intramuscular lipids in old rats than in young ones, notably after DIO (Fig.2). More remarkably, however, variation in lipid metabolites in muscle tissue differed, with a considerable increase in triglycerides (TG) and ceramides and an apparent stability of diacyl-glycerol (DAG) concentration in the muscles of the old obese rats. Previous studies (Hulver *et al*., 2003, 2005; Petersen *et al*., 2003) suggest that insulin resistance development during aging is related to an increase in intramyocellular fatty acid metabolites that may result from an age-associated reduction in mitochondrial oxidative activity. Aging affected the activity of mitochondrial 3-hydroxyacyl-CoA dehydrogenase (HAD), a key enzyme of the β-oxidation cycle, conferring on old rats a reduced ability to oxidize fatty acids entering the muscle cell (Table2). Because balanced proportions of mitochondrial complex activities of mitochondria are required for the normal electron transport chain to function, the calculation of ratios of complex activities is considered as a good index of mitochondrial activity (Miro *et al*., 2000; Zangarelli *et al*., 2006). Tibialis anterior ratios of complex I to complexes II and III were markedly reduced in DIO old rats compared with control old and young obese rats (Table2). Also, ratios of complex II and III to complex IV activities were increased in the same group of old rats. Overall, these findings were linked to a specific 30% reduction in complex I and IV activities in high-fat diet-fed animals. In agreement with the disorganization of the mitochondrial complex activities in DIO old rats, there was a decrease in transcript levels of PGC1α, a transcriptional co-activator playing a multifaceted role in the regulation of mitochondrial energy metabolism and biogenesis. Specifically, PGC1α influences lipid utilization in muscle cells (Anderson *et al*., 2008a). Of note, mitochondrial complexes I and IV are targets of PGC-1 transcriptional activity.

**Table 2:** Alteration in mitochondrial activity in tibialis anterior muscles from young and old rats fed a control or a high-fat diet for 10 weeks

	YLF	YHF	OLF	OHF
Enzyme activities (nmol min^−1^ mg mito proteins^−1^)				
HAD	303 ± 30	327 ± 11	220 ± 13^*^	270 ± 24*
CS	4.1 ± 0.52	4.21 ± 0.20	3.98 ± 0.2	4.64 ± 0.24**
Complex I/II	0.93 ± 0.11	0.81 ± 0.09	0.77 ± 0.04	0.52 ± 0.03*,**
Complex I/III	5.63 ± 1.08	6.16 ± 1.41	7.45 ± 1.70	3.49 ± 0.53*,**
Complex I/IV	0.027 ± 0.004	0.019 ± 0.001	0.019 ± 0.001	0.017 ± 0.001
Complex II/III	6.87 ± 1.19	7.33 ± 1.11	8.57 ± 1.64	6.52 ± 0.77
Complex II/IV	0.024 ± 0.002	0.025 ± 0.002	0.024 ± 0.001	0.032 ± 0.002*,**
Complex III/IV	0.004 ± 0.001	0.004 ± 0.001	0.003 ± 0.001	0.005 ± 0.001*,**
Transcript levels (A.U.)				
TFAM	1.91 ± 0.32	1.72 ± 0.28	1.35 ± 0.26	1.78 ± 0.36
PGC1a	1.17 ± 0.15	1.18 ± 0.16	0.9 ± 0.16	0.78 ± 0.12**
NRF1	0.79 ± 0.21	0.97 ± 0.24	0.65 ± 0.13	0.89 ± 0.16
NRF2a	1.86 ± 0.33	2.01 ± 0.26	1.58 ± 0.27	1.76 ± 0.28

Values ± SEM for eight animals per group.

* *P* < 0.05 age effect (same diet)

** *P* < 0.05 diet effect (same age). Mitochondrial activity was HAD and CS activities, ETC complex activity ratios and mRNA expression of mitochondrial transcriptional factors. YLF, young rats fed a low-fat diet, YHF, young rats fed a high-fat diet, OLF, old rats fed a low-fat diet, OHF, old rats fed a high-fat diet, HAD, 3-hydroxyacyl-CoA dehydrogenase, CS, citrate synthase, TFAM, mitochondrial transcription factor A, PGC1α, PPAR gamma coactivator 1-alpha, NRF1 and 2α, nuclear respiratory factors 1 and 2 alpha.

**Figure 3:**
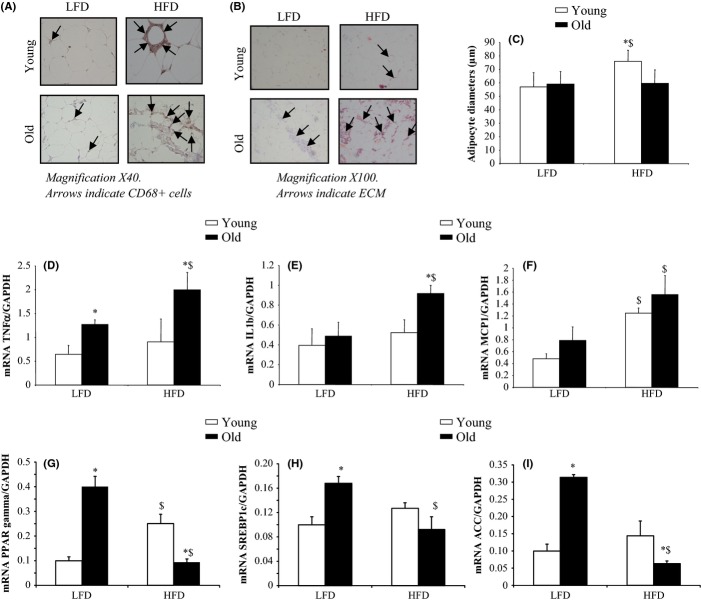
Histological analysis of CD68+ macrophages and extracellular matrix staining, adipocyte diameters and transcript levels of inflammation and adipogenesis markers in abdominal adipose tissue of young and old rats fed a low-fat or a high-fat diet for 10 weeks. Immunohistochemical detection of CD68+ macrophages (A) was performed with the avidin–biotin peroxidase method. Slides of abdominal adipose tissue were stained with picrosirius red to detect areas of fibrosis (B). Adipocyte diameters were measured using perfectimage software (Claravision, France) (C). mRNA transcript levels of TNFα, IL1β MCP1, PPARgamma, SREBP1c, and acetyl-CoA carboxylase (ACC) in abdominal adipose tissue were measured by real-time Q-PCR (D->I). Data are expressed as a ratio of gene expression to GAPDH gene expression. Values ± SEM for eight animals per group. *P < 0.05 age effect (same diet), ^$^*P* < 0.05 diet effect (same age). LFD, low-fat diet; HFD, high-fat diet.

Histological analysis of abdominal adipose tissue revealed increased immune cell accumulation in old rats not only in DIO rats, but also in control animals (Fig.3A). This observation was confirmed when the transcript levels of three major inflammatory intermediates of adipose tissue were analyzed, that is, TNFα, IL1β and MCP1 gene expressions (Fig.3D–F). The morphological changes in adipose tissue were also characterized by lower cell diameter and increased fibrosis in old high-fat rats (Fig.3B,C). The defect in adipogenesis in DIO old rats was confirmed by the decreased expression of key genes of adipogenic enzymes, that is, acetyl-CoA carboxylase (ACC), and transcription factors involved in adipogenesis regulation, that is, PPARgamma and SREBP1c (Fig.3G,H,I).

### Protein synthesis rate is reduced, and translational eIF2-dependent regulation is affected by diet-induced obesity in old rats

**Figure 4:**
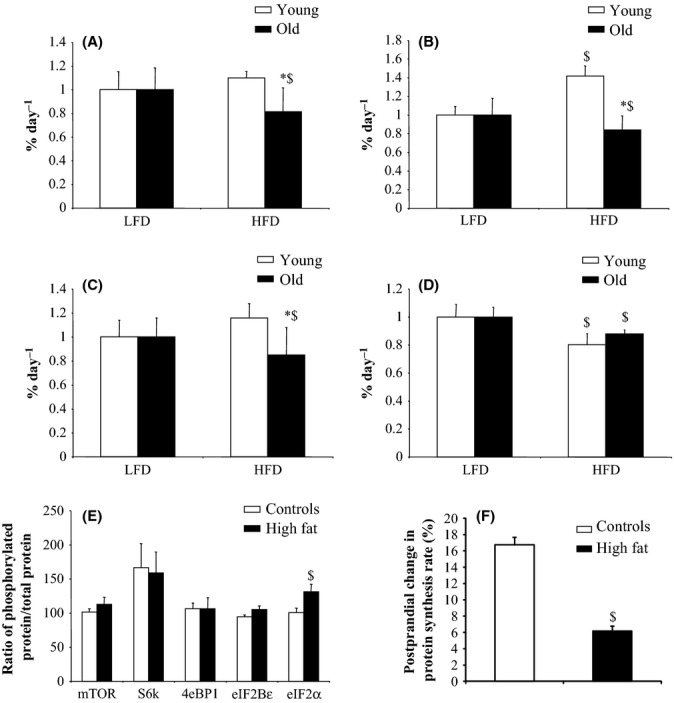
Protein synthesis rate and activation of intracellular pathways related to the regulation of translational initiation in tibialis anterior muscle of young and old rats fed a low-fat or high-fat diet for 10 weeks. Total muscle protein (A) and muscle protein fractions, that is, myosin (B), actin (C), and mitochondrial proteins (D), were isolated, and the rate of protein synthesis was calculated as the ratio of the isotopic enrichment in proteins to the enrichment of the precursor pool of free amino acids. Protein synthesis rates in tibialis anterior muscle are expressed as %/d. The activation of specific intracellular pathways regulating translational initiation in tibialis anterior muscle was assessed in old rats by measuring the phosphorylation state of key intermediates (E). Ratio of phosphorylated protein to total protein content was measured by Western blot. Muscle protein synthesis rate was also measured after food intake in old rats (F). Postprandial protein synthesis rates in tibialis anterior muscle are expressed as % of change compared with postabsorptive values. Values ± SEM for eight animals per group. *P < 0.05 age effect (same diet), ^$^*P* < 0.05 diet effect (same age). LFD, low-fat diet; HFD, high-fat diet.

The synthesis rate of muscle total and mitochondrial proteins and of myosin and actin, two major contractile proteins, fell dramatically in DIO old rats, that is, by ∼18–23%, compared with control old rats (Fig.4A–D). No difference in muscle protein synthesis rate emerged in young rats, except for myosin synthesis, which was significantly increased in obese young rats (+ 30%). Physiologically, the increase in muscle protein synthesis after feeding represents a component of the marked postprandial anabolism which leads to growth and tissue renewal. Here, we observed a lower sensitivity of muscle protein synthesis to nutritional state in DIO old rats (Fig.4F).

Exposure of muscle to growth or stress factors can change protein translation initiation *via* the mTOR and its downstream S6k and 4E-BP1, eIF2B and eIF2α pathways (Kennedy & Kaeberlein, 2009; Kapahi *et al*., 2010; Kaeberlein & Kennedy, 2011).

Total protein expressions and phosphorylation levels were similar between young control and young obese rats (data not shown). Within the old group, the phosphorylation of mTOR, S6k, 4E-BP1 and eIF2B was not affected by DIO. However, protein phosphorylation status of eIF2α was significantly increased in old rats under DIO, a 32% increase in the ratio of phosphorylated eIF2α to total eIF2α being detected in old HF rats (Fig.4E). A 20–30% increase in the phosphorylation of eIF2α frequently suffices to sequester eIF2B in an inactive complex so that recycling of eIF2 is largely inhibited (Kimball *et al*., 2002; Kapahi *et al*., 2010).

### Cultured C2C12 myotubes display reduced protein synthesis and altered eIF2α -dependent translational regulation driven by ceramides

**Figure 5:**
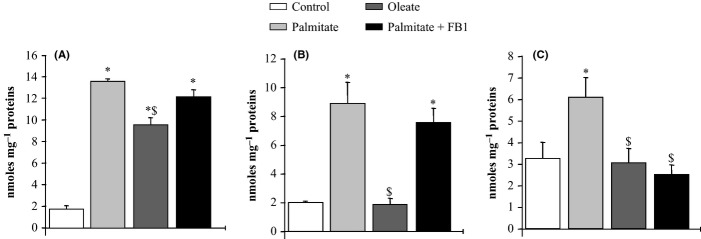
Triglyceride, diacyl-glycerol, and ceramide content in C2C12 myotubes incubated in the presence or absence of fatty acids and of an inhibitor of ceramide synthesis. C2C12 myotubes were incubated in the presence or absence of palmitate (0.375 mm), oleate (0.375 mm), or fumonisin B1 (FB1, 50 μm) for 16 h before lipid extraction. Triglyceride (A), diacyl-glycerol (B), and ceramide (C) concentrations were analyzed by gas-liquid chromatography. Ceramide, diacyl-glycerol, and triglyceride concentrations are expressed as nmoles/mg proteins. Data are from six independent experiments. Values ± SEM. *P < 0.05 vs. control cells, ^$^*P* < 0.05 vs. cells treated with palmitate.

As in previous reports (Chavez *et al*., 2003), myotubes treated with palmitate generated large amounts of reactive lipids, 1.9–7 times more than basal levels for intracellular TG, DAG and ceramide concentrations (Fig.5). The concentration of palmitate used in this study is comparable with that found physiologically and is similar to that used in earlier studies evaluating FFA effects in both immortalized muscle cells (Schmitz-Peiffer *et al*., 1999; Storz *et al*., 1999; Chavez *et al*., 2003) and isolated skeletal muscle strips (Thompson *et al*., 2000). Pretreating C2C12 myotubes with fumonisin B1 (FB1), a fungal toxin that inhibits ceramide synthase, completely prevented the palmitate-induced increase in ceramide levels, but had no effect on TG and DAG accumulation (Fig.5).

**Figure 6:**
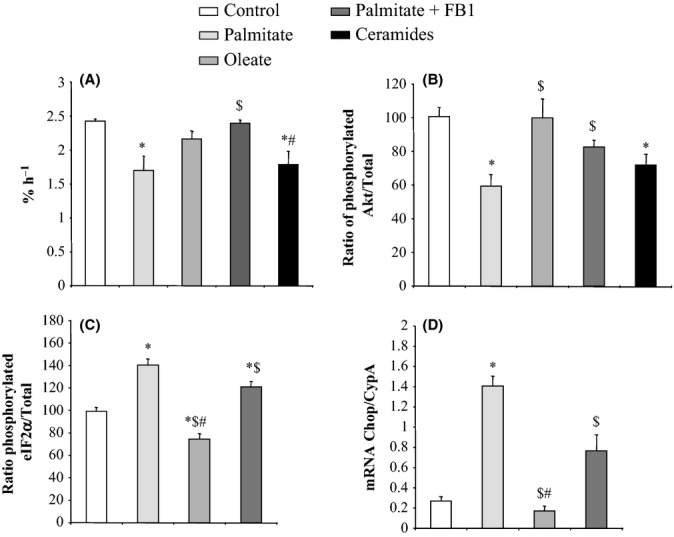
Protein synthesis rate and activation of intracellular pathways related to the regulation of translational initiation in C2C12 myotubes incubated in the presence or absence of fatty acids and of pharmacological mediators of intracellular ceramide content. C2C12 myotubes were incubated in the presence or absence of palmitate (0.375 mm), oleate (0.375 mm), and fumonisin B1 (FB1, 50 μm) for 16 h or C2-ceramide (100 μm) for 30 min before insulin stimulation (100 nm). Proteins were isolated, and protein synthesis rate (A) was determined as in Fig.4. Protein synthesis rates in C2C12 myotubes are expressed as %/h. Data are from six independent experiments. The activation of specific intracellular pathways regulating translational initiation was assessed by measuring the phosphorylation state of key intermediates (B,C). Ratio of phosphorylated protein to total protein content was measured by Western blot. The implication of eIF2α in the regulation of protein translation in C2C12 myotubes treated with fatty acids was confirmed by measuring the mRNA expression of CHOP gene (D), one of the targets of eIF2α. All the data are from six independent experiments. Values ± SEM. *P < 0.05 vs. control cells, ^$^*P* < 0.05 vs. cell treated with palmitate, and ^#^*P* < 0.05 vs. cells treated with palmitate and FB1.

A 30% decrease in protein synthesis rate was seen after incubation of differentiated myotubes with palmitate (Fig.6A). By contrast, after treatment with oleate, C2C12 myotubes generated amounts of proteins equivalent to those from classically cultured control cells. As expected, blocking ceramide metabolism while concomitantly adding palmitate totally prevented palmitate induction of protein synthesis inhibition in myotubes. Finally, the use of short-chain ceramides recapitulated the effects of palmitate on the inhibition of protein synthesis in differentiated muscle cells (Fig.6A).

As reported elsewhere (Merrill, 2002; Chavez *et al*., 2003), palmitate markedly inhibited insulin stimulation of Akt phosphorylation (Fig.6B). By contrast, neither oleate or nor FB1 pretreatment had any effect. Of note, the same effect that we noted with palmitate was observed when ceramide was directly used to treat C2C12 myotubes. Thus, increasing endogenous ceramide levels by an alternative mechanism recapitulated the inhibitory effects of palmitate on Akt phosphorylation state.

In line with our *in vivo* data, the decreased protein synthesis observed in C2C12 myocytes after palmitate incubation was not associated with any difference in the phosphorylation state of mTOR and its substrates S6K and 4E-BP1, compared with the control values (data not shown). The phosphorylation state of eIF2B also appeared to be preserved in C2C12 cells in all conditions (data not shown). By contrast, it is noteworthy that palmitate seemed to upregulate eIF2α phosphorylation level in myotubes, that is, eIF2α was highly phosphorylated in myotubes after palmitate treatment (Fig.6C). A significant protective effect of oleate on eIF2α phosphorylation was seen compared with either palmitate treatment or control cells (Fig.6C). FB1 treatment significantly blunted the effect of palmitate on eIF2α phosphorylation (Fig.6C). In addition to its repressive action of protein translation initiation, the downstream effect of eIF2α is the induction of genes, such as CHOP (CCAAT/enhancer binding protein (C/EBP) homologous protein) (Salminen & Kaarniranta, 2010). Interestingly, in the present work, the evolution of CHOP gene expression after FFA treatments fully corresponded to eIF2α phosphorylation changes in C2C12 myotubes (Fig.6D).

## Discussion

Obesity has been suggested as a risk factor for sarcopenia. However, the underlying pathogenic concept of sarcopenic obesity is mainly based on phenotypical data from clinical observation (Bollheimer *et al*., 2012). The present study describes a rodent animal model which opens up prospects to carry out translational research of sarcopenic obesity in an experimental setting. Our observations support defective plasticity of adipose tissues with age as shown by the lower adipocyte diameter in old DIO rats. These data are evidence of a decline with age in the expandability and storability of adipose tissue under positive energy balance. Accordingly, preadipocytes isolated from old animals accumulate lipids less extensively than cells from young animals, even after weeks in culture under identical conditions (Djian *et al*., 1983; Kirkland *et al*., 1990). In the present study, gene expressions of different adipogenic enzymes, that is, acetyl-CoA carboxylase (ACC), and transcription factors involved in adipogenesis regulation, that is, PPARgamma and SREBP1c, were reduced in the abdominal adipose tissue of old DIO rats. Kirkland *et al*. have proposed that preadipocyte overutilization with aging induces cellular senescence, leading to impaired adipogenesis, failure to sequester lipotoxic fatty acids, and inflammatory cytokine and chemokine generation (Tchkonia *et al*., 2010). As inflammation, for example, TNFα abundance, is anti-adipogenic (Karagiannides *et al*., 2001, 2006; Kirkland *et al*., 2002), we speculated that decreased adipogenesis with aging could result, at least partially, from effects of DIO on pro-inflammatory cytokine production in adipose tissues of old rats. In the present work, increased abdominal adipose tissue inflammation in DIO old animals was associated with a greater number of macrophages, probably due to the increased expression of MCP1. In addition, it was previously reported that under culture conditions in which macrophages were absent, preadipocytes from old animals produced sufficient TNFα to inhibit their own adipogenesis, as evident from TNFα siRNA experiments (Tchkonia *et al*., 2010). Coupled with high numbers of macrophages and pro-inflammatory cytokines, adipose tissue, with its potentially high concentrations of cytotoxic FFAs, may be an especially harsh environment. This environment could damage preadipocytes and increase the production of extracellular matrix components, leading to accelerated adipose tissue fibrosis (Keophiphath *et al*., 2009; Divoux & Clement, 2011), as observed here. It was demonstrated that the co-culture of macrophages with adipocytes caused the macrophages to take on a more alternatively activated (M2) phenotype, which would be expected to promote fibrosis (Spencer *et al*., 2010). Therefore, during aging, the disproportionate accumulation of extracellular matrix components due to high inflammation and lack of remodeling of adipose tissue extracellular matrix may contribute to a failure to expand adipose tissue mass during states of excess caloric intake.

Owing to this effect and to a consequent failure to adequately take up and buffer circulating FFAs, plasma concentration of FFAs was raised in old DIO rats, leading to lipid redistribution and to ectopic lipid accumulation in lean tissues. Previous data revealed accumulating fat depots in skeletal muscle in older subjects, leading to metabolic dysfunction through lipotoxicity and to insulin resistance (Petersen *et al*., 2003; Zoico *et al*., 2010). As in Humans, old control rats displayed fat redistribution, with increased reactive lipid infiltration in skeletal muscle. Interestingly, muscle fat accumulation was strongly enhanced in DIO old rats. In these rats, fatty acids were partly sequestered as less reactive TG within muscle, protecting it against their lipotoxicity. However, a large proportion was converted to ceramides. This subsequent accumulation of lipids within skeletal muscle is known to be associated with several harmful health outcomes such as insulin resistance (Consitt *et al*., 2009). Likewise, insulin resistance was observed in old DIO rats and was related to ectopic fat accumulation. The systemic dysregulation of glucose homeostasis was associated with a decreased ability to upregulate muscle insulin pathway, that is, low Akt phosphorylation state, in old rats fed a high-fat diet. As skeletal muscle is mostly responsible for insulin-stimulated glucose uptake (DeFronzo *et al*., 1985), increased intramuscular fat may be responsible for the decline in glucose tolerance reported in the present work, as previously shown (Pan *et al*., 1997). Several previous studies using different models in animals indicate that chronic exposure to high-fat diet causes severe insulin resistance by increasing fat infiltration inside muscle tissue (Blachnio-Zabielska *et al*., 2010; De Vogel-van den Bosch *et al*., 2010; Ritchie *et al*., 2011). As ceramide synthesis is stimulated by adipocyte-derived cytokines, including TNFα, the systemic and tissular pro-inflammatory state associated with DIO may contribute to muscle lipid infiltration in old rats.

Different intracellular mechanisms have been proposed to explain the ceramide-induced effect on insulin resistance in skeletal muscle. In the present study, palmitate increased ceramide content, inhibiting Akt phosphorylation stimulated by insulin in C2C12 myotubes. Interestingly, the inhibition of *de novo* ceramide synthesis abolished the reduction of Akt phosphorylation in myotubes after palmitate pretreatment, and the use of short-chain ceramides recapitulated the effects of this fatty acid. Therefore, as demonstrated in the model of C2C12 myotubes, ceramide accumulation in muscle tissue was probably one of the direct causes affecting insulin sensitivity in DIO old rats.

High-fat diet and FA accumulation in muscle tissue are also known to cause mitochondrial changes (Bonnard *et al*., 2008; Lambertucci *et al*., 2008). In this study, we have shown that the activity of HAD, a key enzyme for mitochondrial β-oxidation, is affected by age. This observation is evidence in support of a defect in the capacity to oxidize fatty acids and so is probably one of the mechanisms contributing to fatty acid accumulation in skeletal muscle of old DIO rats. Similarly, high-fat diet led to a decrease in both the respiratory chain complex I and IV activities and PGC1α mRNA level in old rats. PGC1α regulates mitochondrial energy metabolism and biogenesis and influences carbohydrate and lipid utilization through activation of members of the nuclear receptor family (Anderson *et al*., 2008a). Old mice overexpressing PGC1α in skeletal muscle were protected against the decline in mitochondrial function and did not develop age-related insulin resistance (Wenz *et al*., 2009). Interestingly, the increased muscle PGC1α expression not only prevents age-associated weight gain and body TG accumulation, but also has a significant beneficial effect on age-associated muscle loss in mice. Therefore, maintenance of muscle lipid content, by preventing the decline in mitochondrial function, improved insulin action and preserved muscle mass during aging (Wenz *et al*., 2009). Interestingly, we previously reported that caloric restriction in old rat is associated with an improved capacity to oxidize lipids in skeletal muscle, that is, increased mitochondrial oxidative activity, together with a significant increase in muscle protein synthesis rate, in muscle mass and in muscle strength (Zangarelli *et al*., 2006; Walrand *et al*., 2011).

In this report, we demonstrate that the provision of a cell-permeable ceramide to C2C12 myotubes results in a significant inhibition of the rate of cellular protein synthesis. This effect was mimicked by palmitate, a physiological substrate for *de novo* ceramide synthesis. In addition, pharmacological intervention to reduce ceramide production restored protein synthesis rate after palmitate treatment in C2C12 myotubes. The results described here point to ceramide as a novel regulator of protein synthesis in C2C12 cells. The rate of protein synthesis is governed by a number of cellular controls, including signaling by the mTOR and eIF2 pathways (Kennedy & Kaeberlein, 2009; Kapahi *et al*., 2010; Kaeberlein & Kennedy, 2011). At variance with *in vitro* observations (Deldicque *et al*., 2010), but in line with *in vivo* data (Khamzina *et al*., 2005), the activity of the mTOR pathway, on which insulin and amino acid availability exert a positive influence, was unaffected in the present study. However, ceramide reduced both protein synthesis and signaling by the eIF2α pathway, that is, upregulated eIF2α phosphorylation state, in C2C12 myotubes. More interestingly, the same inhibition of muscle protein synthesis rate, together with enhanced eIF2α phosphorylation state, was observed in DIO old rats. We therefore claim a causal link between muscle ceramide accumulation, eIF2α hyperphosphorylation and the reduced rate of muscle protein synthesis induced by high-fat intake in old rats. eIF2α is phosphorylated in response to environmental stresses to alleviate cell injury. It is well known that the phosphorylation of the α subunit of eIF2 reduces global translation, allowing cells to conserve resources and initiate a reconfiguration of gene expression to effectively manage stress conditions (Wek *et al*., 2006). Accompanying this general protein synthesis control, eIF2α phosphorylation induces translation of selected mRNAs, such as those encoding for transcription factors, for example, CHOP, that assist the regulation of genes involved in metabolism. Interestingly, the transcript level of the CHOP gene was increased here, bringing additional evidence for the participation of this specific pathway in the effect of ceramide on the rate of cellular protein synthesis in old DIO rats.

Several studies (Anderson *et al*., 2008b; Sitnick *et al*., 2009) have demonstrated that postprandial activation of muscle protein synthesis and load-induced skeletal muscle hypertrophy are impaired in high-fat-fed animals. The present study demonstrates that muscle protein synthesis becomes less sensitive to nutritional state in old high-fat-fed rats. Taken together, these previous data and the present study highlight that DIO in rodents appears to alter normal protein dynamics in anabolic conditions, that is, food intake and muscle loading, and during aging. As shown here, this effect seems to be explained by a decrease in the activation of Akt pathway likely associated with muscle lipid infiltration. As a consequence, a high-fat diet could accelerate the effect of aging on muscle mass and function, as well as impede recovery from injuries.

Our study bears certain limitations. First, the data for immunohistochemistry are only descriptive. It would have been interesting to quantify the histological observations by measuring the number of activated macrophages and the optical density of areas positive for fibrosis. However, the adipose tissue sections observed in this study were reproducible with a systematic increase in macrophage infiltration and areas of fibrosis in old rats fed a high-fat diet. Second, several environmental factors may differ between young and old rats and normal diet and high-fat diet rats, such as the level of physical activity. Physical activity might probably differ between the young and old animals and between the DIO and control groups due to heavier body weight of the DIO animals. The individual physical activity of the animals should be taken into account in further studies as a possible confounder (Bollheimer *et al*., 2012). Third, it would have been informative to evaluate the potential interest of nutritional strategies able to counteract muscle lipotoxicity in old DIO rats, for example, short-term caloric restriction, to maintain or improve muscle protein anabolism. Fourth, this study is an exploratory study conducted in rodents. Little evidence in the literature suggests that the same phenomena occur in older Humans and explain the appearance of sarcopenic obesity. Clinical studies are needed to assess the potential involvement of adipogenic capacities and muscle lipotoxicity in the pathogenesis of sarcopenic obesity in Humans.

In conclusion, like Humans, rats exhibit ectopic fat deposition with aging, in particular when the DIO model is used. Therefore, the old high-fat-fed rat proved to be feasible for an experimental study of sarcopenic obesity and provided explicit pathogenic connecting points. The decreased ability of adipose tissue to store lipids contributes to the accumulation of intramuscular lipids, which place a lipid burden on mitochondria and may create a disconnect between metabolic and regulating pathways in skeletal muscles of old rats. Although the precise connection between mitochondrial activity and insulin resistance is still debated, our data demonstrate that the increased accumulation of lipid metabolism by-products is linked to impaired insulin action in palmitate-treated C2C12 myotubes and in old rats fed a high-fat diet. Additionally, the present study reveals that intramuscular lipid deposition in old DIO rats, together with palmitate-mediated lipid accumulation in C2C12 cells, affects protein synthesis rate *via* eIF2α phosphorylation. The use of pharmacological inhibitors of ceramide production or the use of ceramide confirms these effects, showing that the harmful action of reactive lipids on muscle protein synthesis during aging is probably driven by ceramide accumulation inside muscle cells. Therefore, a decrease in the ability of adipose tissue to respond to DIO with age could adversely affect protein metabolism in skeletal muscle due to the toxicity of lipid species and thereby accelerate the effects of aging, that is, sarcopenia. This process may contribute to the loss of muscle function observed in sarcopenic obese patients.

## Experimental procedures

### Experimental design

The animal facilities and protocol were approved by the local animal ethical committee. Male Wistar rats aged 5 months (young) and 25 months (old) were purchased from Janvier (Le Genest St Isle, France).

After 1-week acclimatization with a standard diet, the rats were randomly divided into four groups as follows: young rats fed a standard diet (YC, *n* = 8), young rats fed a high-energy high-fat diet (YHF, *n* = 8), old rats fed a standard diet (OC, *n* = 8), and old rats fed a high-energy high-fat diet (OHF, *n* = 8). Additional rats were used to study the postprandial effect of high-fat diet on muscle protein synthesis rate. Experimental diet composition is given as Table S1 (Supporting information). At the end of the 10 weeks of diet, the rats were anesthetized and killed by a heart blood puncture. The muscles (soleus, tibialis anterior, plantaris, gastrocnemius, extensor digitorum longus, quadriceps) and adipose tissues were removed, weighed, and frozen in liquid nitrogen and stored at −80 °C until analysis.

### Muscle cell culture

Differentiated C2C12 myotubes were cultured for 16 h with palmitic acid (0.375 mm) and oleic acid (0.375 mm) in the presence or absence of fumonisin B1 (FB1), a specific inhibitor of ceramide synthase. To study the specific effect of ceramides, myotubes were incubated in the presence of short-chain ceramides (C2-ceramides, 100 μm, 30 min). Aliquots of cell lysates were saved to isolate protein content in each sample. Protein synthesis rate was evaluated as described below.

### Analysis

#### Measurement of *in vivo* and *in vitro* protein synthesis rates

A 50-mg piece of tibialis anterior or C2C12 cell proteins was used for the isolation of total protein fraction, myosin, actin, and mitochondrial proteins as previously described (Zangarelli *et al*., 2004, 2006). Isotopic enrichment in proteins was measured by gas chromatography-combustion-isotope ratio mass spectrometry (μGas System, Fisons Instruments, VG Isotech, Middlewich, UK). Isotopic enrichments in tissue fluid were assessed using gas chromatography-mass spectrometry (Hewlett-Packard 5971A; Hewlett-Packard Co., Palo Alto, CA, USA) and were used as precursor pool enrichment for the calculations of the fractional synthesis rates (FSR).

FSR of proteins were calculated using the equation: 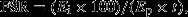
where *E*_i_ is the enrichment as atom percentage excess of tracer from proteins at time *t* (minus basal enrichment), *E*_p_ is the mean enrichment in the precursor pool (tissue fluid), and *t* is the incorporation time in hours. Data are expressed as %/day for *in vivo* experiments and as %/hour for *in vitro* experiments.

#### Intraperitoneal glucose tolerance test

Intraperitoneal glucose tolerance test (IPGTT) was performed 2 weeks before slaughtering and 12 h after food withdrawal. Glucose solution was injected into the peritoneal cavity (1 g glucose/kg body weight). Blood samples were collected before glucose administration and 15, 30, 60, and 120 min later. Blood glucose concentration was measured using a glucometer (coefficient of variation of the assay: 5%; OneTouch Glucotouch, LifeScan, Milpitas, CA, USA). Serum insulin levels were measured with an Elisa kit (CV: 2%; Rat/Mouse Insulin Elisa, Linco Research, St. Charles, MO, USA). Insulin sensitivity was estimated by the product of the area under the curve (AUC) of the glucose and the AUC of insulin.

#### Biochemical and hormonal assay in blood and tissues

Fasting plasma concentrations of total cholesterol (CV: 3%), triglycerides (CV: 4%), and FFA (CV: 4%) were measured on an automated analyzer (Konelab 20; Thermo Electron, Waltham, MA). Fasting plasma concentrations of sTNF-R1 (CV: 5%), sTNF-R2 (CV: 4%), leptin (CV: 5%), and adiponectin (CV: 5%) were measured by enzyme-linked immunosorbent assay (ELISA, BioCat, Heidelberg, Germany).

#### Western blot analysis

Denaturated proteins (50 μg) from tibialis anterior or C2C12 myotubes were separated by SDS-PAGE on a 10% or a 4–12% gradient polyacrylamide gel and transferred to a polyvinylidene membrane (Millipore). Immunoblots were probed with primary antibodies: antiphospho protein kinase B (Akt, Ser473), antitotal Akt, antiphospho mTOR (Ser2448), antitotal mTOR, antiphospho S6 kinase (S6k, Thr389) and antitotal S6k, antiphospho eukaryotic initiation factor 4-binding protein 1 (4E-BP1, Ser65), antitotal 4E-BP1, antiphospho eukaryotic initiation factor 2-α (eIF2-α, Ser51), antitotal eIF2-α, antiphospho eukaryotic initiation factor 2b-ε (eIF2b-ε Ser9), and antitotal eIF2b-ε. All the antibodies were purchased from Cell Signaling Technology (Ozyme distributor, Saint-Quentin-en-Yvelines, France), except for antiphospho eIF2b-ε (Genetex, Emmedex distributor, Mundolsheim, France). Immunoblots were incubated with a horseradish peroxidase-conjugated secondary antibody (DAKO, Trappes, France). The immune reactive strips were visualized by chemoluminescence (ECL Western Blotting Substrate, Pierce, USA). Luminescent visualization of the secondary antibodies was performed using Biomax light film (Kodak Scientific, New Haven, CT, USA). The activation states were evaluated by the ratio of phosphorylated protein to total protein expression.

#### Real-time quantitative RT–PCR analysis

Total RNAs were extracted from 50 mg of tibialis anterior, abdominal adipose tissue, or C1C12 cell lysate with 1 ml of TRIzol (TRIzol® Reagent, Invitrogen, Carlsbad, CA, USA). DNAc probes were obtained using SuperScript II H-RNase kit (Invitrogen). Relative RNA levels were determined by analyzing the changes in SYBR green I fluorescence (Kit Fast Start DNA Master SYBR Green I, Roche, Rotkreuz, Switzerland) during PCR according to the manufacturer's instructions. The expression of GAPDH, HPRT, and Cyp A genes was amplified, and the results were used for normalization of gene expressions in adipose tissue, skeletal muscle, and C2C12 myotubes, respectively.

#### Intramuscular and intracellular lipid contents

Lipids were extracted from tibilias anterior or C2C12 cell lysate according to Bligh and Dyer (Bligh & Dyer, 1959) in the presence of the internal standards. Lipids were analyzed by gas-liquid chromatography on a FOCUS Thermo Electron system using a Zebron-1 Phenomenex fused silica capillary column (5 m × 0.32 mm i.d, 0.50 μm film thickness).

#### Immunohistochemical detection of CD68+ macrophages, fibrosis visualization, and adipocyte size measurement in abdominal adipose tissue

Immunohistochemical detection of macrophages using CD68 (Neomarker Microm, Francheville, France) was performed with the avidin–biotin peroxidase (ABC) method. Processed slide images were acquired by a microscope-camera system at ×40 magnification (Nikon, France).

Slides of abdominal adipose tissue were also stained with picrosirius red. Fibrosis analysis was performed using Alphalys platform (histolab software, Plaisir, France) at ×100 magnification with constant color thresholds.

Adipose tissue samples were fixed overnight at 4 °C in 4% paraformaldehyde and processed for standard paraffin embedding. Sections 5 μm thick were stained as described below and examined under a Zeiss 20 Axiostar Plus microscope (Zeiss, Germany). Digital images were captured by a camera (triCCD, Sony, France). Adipocyte diameters were measured using perfectimage Software (Claravision, France).

Two sections per sample were analyzed. The two sections were spaced by 25 micrometers, that is, five sections spacing. The observation was made blindly by two independent observers.

#### Mitochondrial enzyme activities and gene expression

Citrate synthase (CS), 3-hydroxyacyl-CoA dehydrogenase (HAD), and the activities of complexes I to IV were assayed spectrophotometrically in the mitochondrial suspension from tibialis anterior (Zangarelli *et al*., 2004, 2006). All measures were performed in triplicate. Enzyme activities were expressed in nmol min^−1^ mg^−1^ mitochondrial proteins or as activity ratios (Zangarelli *et al*., 2004, 2006).

Abundance of selected mRNAs in muscle mitochondria was measured with the real-time quantitative PCR system as described above.

### Statistical analysis

All data are presented as means *±* SEM. A two-way analysis of variance (ANOVA) was performed to test the effect of the experimental nutritional conditions and the effect of age. When a significant effect was detected, an *a posteriori* Fisher test was applied to locate pairwise differences between groups. The data were normally distributed. statview software (version 4.02; Abacus Concepts, Berkeley, CA, USA) was used for the statistical analyses. Values of *P *<* *0.05 were considered significant.

## Author contributions

Nicolas Tardif and Jerome Salles participated in the definition of the study design and in the analysis of the samples. Christelle Guillet helped in designing the study. Joan Tordjman analyzed inflammation and fibrosis markers in adipose tissues. Sophie Reggio analyzed inflammation and fibrosis markers in adipose tissues. Jean-François Landrier analyzed gene expression in adipose tissue. Christophe Giraudet and Véronique Patrac participated in the analyses. Justine Bertrand-Michel analyzed lipid content in skeletal muscles and C2C12 cells. Carole Migné performed the mass spectrometry analyses. Jean-Michel Chardigny participated in the definition of the study design. Yves Boirie participated in the definition of the study design. Stéphane Walrand designed the study and participated in the analysis of the samples and data and drafted the paper.

## Funding

This study was funded by the French National Research Agency (ANR, LIPAGE Project).

## Conflict of interest

No conflict of interest is related to this study.

## Supporting Information

**Table S1.** Diet composition. **Table S2.** Primer sequences for the quantitative analysis of gene expression.

## References

[b1] Anderson RM, Barger JL, Edwards MG, Braun KH, O'Connor CE, Prolla TA, Weindruch R (2008a). Dynamic regulation of PGC-1alpha localization and turnover implicates mitochondrial adaptation in calorie restriction and the stress response. Aging Cell.

[b2] Anderson SR, Gilge DA, Steiber AL, Previs SF (2008b). Diet-induced obesity alters protein synthesis: tissue-specific effects in fasted versus fed mice. Metabolism.

[b3] Blachnio-Zabielska A, Baranowski M, Zabielski P, Gorski J (2010). Effect of high fat diet enriched with unsaturated and diet rich in saturated fatty acids on sphingolipid metabolism in rat skeletal muscle. J. Cell. Physiol.

[b4] Bligh EG, Dyer WJ (1959). A rapid method of total lipid extraction and purification. Can. J. Biochem. Physiol.

[b5] Bollheimer LC, Buettner R, Pongratz G, Brunner-Ploss R, Hechtl C, Banas M, Singler K, Hamer OW, Stroszczynski C, Sieber CC, Fellner C (2012). Sarcopenia in the aging high-fat fed rat: a pilot study for modeling sarcopenic obesity in rodents. Biogerontology.

[b6] Bonnard C, Durand A, Peyrol S, Chanseaume E, Chauvin MA, Morio B, Vidal H, Rieusset J (2008). Mitochondrial dysfunction results from oxidative stress in the skeletal muscle of diet-induced insulin-resistant mice. J. Clin. Invest.

[b7] Chavez JA, Knotts TA, Wang LP, Li G, Dobrowsky RT, Florant GL, Summers SA (2003). A role for ceramide, but not diacylglycerol, in the antagonism of insulin signal transduction by saturated fatty acids. J. Biol. Chem.

[b8] Consitt LA, Bell JA, Houmard JA (2009). Intramuscular lipid metabolism, insulin action, and obesity. IUBMB Life.

[b9] De Vogel-van den Bosch J, Hoeks J, Timmers S, Houten SM, van Dijk PJ, Boon W, Van Beurden D, Schaart G, Kersten S, Voshol PJ, Wanders RJ, Hesselink MK, Schrauwen P (2010). The effects of long- or medium-chain fat diets on glucose tolerance and myocellular content of lipid intermediates in rats. Obesity (Silver Spring).

[b10] DeFronzo RA, Gunnarsson R, Bjorkman O, Olsson M, Wahren J (1985). Effects of insulin on peripheral and splanchnic glucose metabolism in noninsulin-dependent (type II) diabetes mellitus. J. Clin. Invest.

[b11] Deldicque L, Cani PD, Philp A, Raymackers JM, Meakin PJ, Ashford ML, Delzenne NM, Francaux M, Baar K (2010). The unfolded protein response is activated in skeletal muscle by high-fat feeding: potential role in the downregulation of protein synthesis. Am. J. Physiol. Endocrinol. Metab.

[b12] Delmonico MJ, Harris TB, Visser M, Park SW, Conroy MB, Velasquez-Mieyer P, Boudreau R, Manini TM, Nevitt M, Newman AB, Goodpaster BH, Health, Aging, and Body (2009). Longitudinal study of muscle strength, quality, and adipose tissue infiltration. Am. J. Clin. Nutr.

[b13] Divoux A, Clement K (2011). Architecture and the extracellular matrix: the still unappreciated components of the adipose tissue. Obes. Rev.

[b14] Djian P, Roncari AK, Hollenberg CH (1983). Influence of anatomic site and age on the replication and differentiation of rat adipocyte precursors in culture. J. Clin. Invest.

[b15] Goodpaster BH, He J, Watkins S, Kelley DE (2001). Skeletal muscle lipid content and insulin resistance: evidence for a paradox in endurance-trained athletes. J. Clin. Endocrinol. Metab.

[b16] Guillet C, Delcourt I, Rance M, Giraudet C, Walrand S, Bedu M, Duche P, Boirie Y (2009). Changes in basal and insulin and amino acid response of whole body and skeletal muscle proteins in obese men. J. Clin. Endocrinol. Metab.

[b17] Henique C, Mansouri A, Fumey G, Lenoir V, Girard J, Bouillaud F, Prip-Buus C, Cohen I (2010). Increased mitochondrial fatty acid oxidation is sufficient to protect skeletal muscle cells from palmitate-induced apoptosis. J. Biol. Chem.

[b18] Hulver MW, Berggren JR, Cortright RN, Dudek RW, Thompson RP, Pories WJ, MacDonald KG, Cline GW, Shulman GI, Dohm GL, Houmard JA (2003). Skeletal muscle lipid metabolism with obesity. Am. J. Physiol. Endocrinol. Metab.

[b19] Hulver MW, Berggren JR, Carper MJ, Miyazaki M, Ntambi JM, Hoffman EP, Thyfault JP, Stevens R, Dohm GL, Houmard JA, Muoio DM (2005). Elevated stearoyl-CoA desaturase-1 expression in skeletal muscle contributes to abnormal fatty acid partitioning in obese humans. Cell Metab.

[b20] Kaeberlein M, Kennedy BK (2011). Hot topics in aging research: protein translation and TOR signaling, 2010. Aging Cell.

[b21] Kapahi P, Chen D, Rogers AN, Katewa SD, Li PW, Thomas EL, Kockel L (2010). With TOR, less is more: a key role for the conserved nutrient-sensing TOR pathway in aging. Cell Metab.

[b22] Karagiannides I, Tchkonia T, Dobson DE, Steppan CM, Cummins P, Chan G, Salvatori K, Hadzopoulou-Cladaras M, Kirkland JL (2001). Altered expression of C/EBP family members results in decreased adipogenesis with aging. Am. J. Physiol. Regul. Integr. Comp. Physiol.

[b23] Karagiannides I, Kokkotou E, Tansky M, Tchkonia T, Giorgadze N, O'Brien M, Leeman SE, Kirkland JL, Pothoulakis C (2006). Induction of colitis causes inflammatory responses in fat depots: evidence for substance P pathways in human mesenteric preadipocytes. Proc. Natl Acad. Sci. USA.

[b24] Katsanos CS, Mandarino LJ (2011). Protein metabolism in human obesity: a shift in focus from whole-body to skeletal muscle. Obesity.

[b25] Kennedy BK, Kaeberlein M (2009). Hot topics in aging research: protein translation, 2009. Aging Cell.

[b26] Keophiphath M, Achard V, Henegar C, Rouault C, Clement K, Lacasa D (2009). Macrophage-secreted factors promote a profibrotic phenotype in human preadipocytes. Mol. Endocrinol.

[b27] Khamzina L, Veilleux A, Bergeron S, Marette A (2005). Increased activation of the mammalian target of rapamycin pathway in liver and skeletal muscle of obese rats: possible involvement in obesity-linked insulin resistance. Endocrinology.

[b28] Kim JY, van de Wall E, Laplante M, Azzara A, Trujillo ME, Hofmann SM, Schraw T, Durand JL, Li H, Li G, Jelicks LA, Mehler MF, Hui DY, Deshaies Y, Shulman GI, Schwartz GJ, Scherer PE (2007). Obesity-associated improvements in metabolic profile through expansion of adipose tissue. J. Clin. Invest.

[b29] Kimball SR, Farrell PA, Jefferson LS (2002). Invited Review: Role of insulin in translational control of protein synthesis in skeletal muscle by amino acids or exercise. J. Appl. Physiol.

[b30] Kirkland JL, Hollenberg CH, Gillon WS (1990). Age, anatomic site, and the replication and differentiation of adipocyte precursors. Am. J. Physiol.

[b31] Kirkland JL, Tchkonia T, Pirtskhalava T, Han J, Karagiannides I (2002). Adipogenesis and aging: does aging make fat go MAD?. Exp. Gerontol.

[b32] Lambertucci RH, Hirabara SM, Silveira Ldos R, Levada-Pires AC, Curi R, Pithon-Curi TC (2008). Palmitate increases superoxide production through mitochondrial electron transport chain and NADPH oxidase activity in skeletal muscle cells. J. Cell. Physiol.

[b33] Mazzali G, Di Francesco V, Zoico E, Fantin F, Zamboni G, Benati C, Bambara V, Negri M, Bosello O, Zamboni M (2006). Interrelations between fat distribution, muscle lipid content, adipocytokines, and insulin resistance: effect of moderate weight loss in older women. Am. J. Clin. Nutr.

[b34] Merrill AH (2002). *De novo* sphingolipid biosynthesis: a necessary, but dangerous, pathway. J. Biol. Chem.

[b35] Miro O, Casademont J, Casals E, Perea M, Urbano-Marquez A, Rustin P, Cardellach F (2000). Aging is associated with increased lipid peroxidation in human hearts, but not with mitochondrial respiratory chain enzyme defects. Cardiovasc. Res.

[b36] Pan DA, Lillioja S, Kriketos AD, Milner MR, Baur LA, Bogardus C, Jenkins AB, Storlien LH (1997). Skeletal muscle triglyceride levels are inversely related to insulin action. Diabetes.

[b37] Petersen KF, Befroy D, Dufour S, Dziura J, Ariyan C, Rothman DL, DiPietro L, Cline GW, Shulman GI (2003). Mitochondrial dysfunction in the elderly: possible role in insulin resistance. Science.

[b38] Ritchie IR, Gulli RA, Stefanyk LE, Harasim E, Chabowski A, Dyck DJ (2011). Restoration of skeletal muscle leptin response does not precede the exercise-induced recovery of insulin-stimulated glucose uptake in high-fat-fed rats. Am. J. Physiol. Regul. Integr. Comp. Physiol.

[b39] Ryan AS, Nicklas BJ (1999). Age-related changes in fat deposition in mid-thigh muscle in women: relationships with metabolic cardiovascular disease risk factors. Int. J. Obes. Relat. Metab. Disord.

[b40] Salminen A, Kaarniranta K (2010). ER stress and hormetic regulation of the aging process. Ageing Res. Rev.

[b41] Schmitz-Peiffer C, Craig DL, Biden TJ (1999). Ceramide generation is sufficient to account for the inhibition of the insulin-stimulated PKB pathway in C2C12 skeletal muscle cells pretreated with palmitate. J. Biol. Chem.

[b42] Sitnick M, Bodine SC, Rutledge JC (2009). Chronic high fat feeding attenuates load-induced hypertrophy in mice. J. Physiol.

[b43] Slawik M, Vidal-Puig AJ (2007). Adipose tissue expandability and the metabolic syndrome. Genes Nutr.

[b44] Spencer M, Yao-Borengasser A, Unal R, Rasouli N, Gurley CM, Zhu B, Peterson CA, Kern PA (2010). Adipose tissue macrophages in insulin-resistant subjects are associated with collagen VI and fibrosis and demonstrate alternative activation. Am. J. Physiol. Endocrinol. Metab.

[b45] Storz P, Doppler H, Wernig A, Pfizenmaier K, Muller G (1999). Cross-talk mechanisms in the development of insulin resistance of skeletal muscle cells palmitate rather than tumour necrosis factor inhibits insulin-dependent protein kinase B (PKB)/Akt stimulation and glucose uptake. Eur. J. Biochem.

[b46] Tchkonia T, Morbeck DE, Von Zglinicki T, Van Deursen J, Lustgarten J, Scrable H, Khosla S, Jensen MD, Kirkland JL (2010). Fat tissue, aging, and cellular senescence. Aging Cell.

[b47] Thompson AL, Lim-Fraser MY, Kraegen EW, Cooney GJ (2000). Effects of individual fatty acids on glucose uptake and glycogen synthesis in soleus muscle *in vitro*. Am. J. Physiol. Endocrinol. Metab.

[b48] Tiraby C, Tavernier G, Capel F, Mairal A, Crampes F, Rami J, Pujol C, Boutin JA, Langin D (2007). Resistance to high-fat-diet-induced obesity and sexual dimorphism in the metabolic responses of transgenic mice with moderate uncoupling protein 3 overexpression in glycolytic skeletal muscles. Diabetologia.

[b49] Walrand S, Zangarelli A, Guillet C, Salles J, Soulier K, Giraudet C, Patrac V, Boirie Y (2011). Effect of fast dietary proteins on muscle protein synthesis rate and muscle strength in *ad libitum*-fed and energy-restricted old rats. Br. J. Nutr.

[b50] Wek RC, Jiang HY, Anthony TG (2006). Coping with stress: eIF2 kinases and translational control. Biochem. Soc. Trans.

[b51] Wenz T, Rossi SG, Rotundo RL, Spiegelman BM, Moraes CT (2009). Increased muscle PGC-1alpha expression protects from sarcopenia and metabolic disease during aging. Proc. Natl Acad. Sci. USA.

[b52] Zangarelli A, Walrand S, Guillet C, Gachon P, Rousset P, Giraudet C, Picard B, Boirie Y (2004). Centrifugation-based isolation of myosin for measurement of its synthesis rate in small muscle samples. Anal. Biochem.

[b53] Zangarelli A, Chanseaume E, Morio B, Brugère C, Mosoni L, Rousset P, Giraudet C, Patrac V, Gachon P, Boirie Y, Walrand S (2006). Synergistic effects of caloric restriction with maintained protein intake on skeletal muscle performance in 21-month-old rats: a mitochondria-mediated pathway. FASEB J.

[b54] Zoico E, Rossi A, Di Francesco V, Sepe A, Olioso D, Pizzini F, Fantin F, Bosello O, Cominacini L, Harris TB, Zamboni M (2010). Adipose tissue infiltration in skeletal muscle of healthy elderly men: relationships with body composition, insulin resistance, and inflammation at the systemic and tissue level. J. Gerontol. A Biol. Sci. Med. Sci.

