# Supplementary material for: Muscle ectopic fat deposition contributes to anabolic resistance in obese sarcopenic old rats through eIF2α activation
Source: Aging Cell. 2014 Aug 19;13(6):1001–11. doi: 10.1111/acel.12263 (PMC4326920; doi:10.1111/acel.12263)
Supplement: Supplementary file 1 — Table S1. Diet composition. Table S2. Primer sequences for the quantitative analysis of gene expression. [file acel0013-1001-sd1.docx]

**Supplemental data**: diet composition

| *% of total energy intake* | **Low-fat diet** | **High-fat diet** |
| --- | --- | --- |
| Casein  Cornstarch  Sucrose  Sunflower oil  Rapeseed oil  Lard | 17.4  68.7  -  6.9  6.9  - | 17.4  18.9  18.9  6.7  6.7  31.4 |

Diets provided approximately 370 kJ per day to the standard diet groups (YC and OC) and 33% more energy to the high-fat groups (YHF, OHF), have the same amount of nitrogen, cellulose (48g/kg) and vitamins and minerals (72 g/kg).

**Supplemental data**: primer sequences for the quantitative analysis of gene expression

|  | **Gene name** | **Forward and reverse primers** | |  |
| --- | --- | --- | --- | --- |
| **Skeletal**  **Muscle** | NRF1  (Nuclear respiratory factor 1) | | *For 5’-*TTATTCTGCTGTGGCTGATGG*-3’*  *Rev 5’-*CCTCTGATGCTTGCGTCGTCT*-3’* | |
|  | NRF2α  (Nuclear respiratory factor 2 alpha) | | *For 5’-*CACCACACTCAACATTTCGG*-3’*  *Rev 5’-*TGGAGATGCAATTGCTCAGT*-3’* | |
|  | PGC1α  (Peroxisome proliferator-activated receptor gamma coactivator 1 alpha) | | *For 5’-*AGTTTTTGGTGAAATTGAGGAAT*-3’*  *Rev 5’-* TCATACTTGCTCTTGGTGGAAGC*-3’* | |
|  | TFAM  (Mitochondrial transcription factor A) | | *For 5’-* CTGCTTTTCATCATGAGACAG*-3’*  *Rev 5’-*GAAAGCACAAATCAAGAGGAG*-3’* | |
|  | HPRT  (Hypoxanthine-guanine phosphoribosyltransferase) | | *For 5’-*AGTTGAGAGATCATCTCCAC*-3’*  *Rev 5’-*TTGCTGACCTGCTGGATTAC*-3’* | |
| **Adipose**  **tissue** | IL1β  (Interleukin-1 beta)  TNFα  (Tumor necrosis factor alpha)  MCP1  (Monocyte chemotactic protein 1)  PPARgamma  (Peroxisome proliferator-activated receptor gamma)  SREBP1c  (Sterol regulatory element binding protein-1)  ACC  (Acetyl-CoA carboxylase)  GAPDH  (Glyceraldehyde 3-phosphate dehydrogenase) | | *For 5’-*CACCTTCTTTTCCTTCATCTTTG*-3’*  *Rev 5’-*GTCGTTGCTTGTCTCTCCTTGTA*-3’*  *For 5’-*ACTCGAGTGACAAGCCCGTA*-3’*  *Rev 5’-*TGGAAGACTCCTCCCAGGT*-3’*  *For 5’-*GCTGCTACTCATTCACTGGCAA*-3’*  *Rev 5’-* TGCTGCTGGTGATTGTCTTGTA*-3’*  *For 5’-*aatggcatctctgtgtcaacc*-3’*  *Rev 5’-*ggtgaaactctgggagatcct*-3’*  *For 5’-*tgcgcaagacagcagattta*-3’*  *Rev 5’-*acaagattgtggagctcaagg*-3’*  *For 5’-*caggctaccatgccaatctc*-3’*  *Rev 5’-*gatgatcaaggccagcttgt*-3’*  *For 5’-* GAACATCATCCCTGCATCCA*-3’*  *Rev 5’-* CCAGTGAGCTTCCCGTTCA*-3’* | |
| **C2C12 myotubes** | Chop  (CCAAT-enhancer-binding protein homologous protein)  Cyp A  (Cyclophilin A) | | *For 5’-*CCTAGCTTGGCTGACAGAGG*-3’*  *Rev 5’-*CTGCTCCTTCTCCTTCATGC*-3’*  *For 5’-*TATCTGCACTGCCAAGACTGAGTG*-3’*  *Rev 5’-*CTTCTTGCTGGTCTTGCCATTCC*-3’* | |
